# SimulScan and Partial Least Squares: Visualizing Swallowing Through Functional and Dynamic Imaging Correlations

**DOI:** 10.1002/mrm.70481

**Published:** 2026-06-21

**Authors:** Bradley P. Sutton, Anthony Bosshardt, Ching‐Hsuan Peng, Ololade T. Adetula, Jiyoon Kim, Riwei Jin, Vaishnavi Krishna, William G. Pearson, Zhongming Liu, Georgia A. Malandraki

**Affiliations:** ^1^ Beckman Institute for Advanced Science and Technology University of Illinois Urbana Champaign Urbana Illinois USA; ^2^ Department of Bioengineering Grainger College of Engineering, University of Illinois Urbana Champaign Urbana Illinois USA; ^3^ Carle Illinois College of Medicine University of Illinois Urbana Champaign Urbana Illinois USA; ^4^ Biohub Chicago Chicago Illinois USA; ^5^ Department of Speech, Language, & Hearing Sciences, College of Health and Human Sciences Purdue University West Lafayette Indiana USA; ^6^ Coordinated Science Lab University of Illinois Urbana Champaign Urbana Illinois USA; ^7^ Department of Biomedical Sciences Edward Via College of Osteopathic Medicine Auburn Alabama USA; ^8^ Department of Biomedical Engineering University of Michigan Ann Arbor Ann Arbor Michigan USA; ^9^ Department of Electrical and Computer Engineering University of Michigan Ann Arbor Ann Arbor Michigan USA; ^10^ Department of Speech & Hearing Science, College of Applied Health Sciences University of Illinois Urbana Champaign Champaign Illinois USA

**Keywords:** dynamic MRI, functional analysis, functional MRI, swallowing

## Abstract

**Purpose:**

Swallowing involves the precise coordination of muscles and brain areas and can be disrupted in a variety of neurological conditions. Current methods to visualize swallowing cannot examine both the biomechanics and brain activity associated with specific swallowing events. An updated version of a pulse sequence that simultaneously samples BOLD‐based fMRI and dynamic imaging (called SimulScan) is introduced that provides higher quality and faster dynamic imaging, enabling data‐driven analysis of swallowing function through a partial least squares (PLS) analysis.

**Methods:**

Integrating updated dynamic imaging approaches, SimulScan achieved dynamic MRI at 23.75 frames per second with a 30 cm field of view with BOLD fMRI at a 1.6 s TR. Five subjects were scanned with SimulScan twice and with videofluoroscopy to compare the preliminary reliability of measuring swallowing biomechanics using computational analysis of swallowing mechanics (CASM) and the test–retest relationship in correlated functional and dynamic components of PLS.

**Results:**

High reliability of biomechanical measures of swallowing was achieved across the two SimulScan runs with CASM (*r* = 0.891; *p* < 0.0001) and between SimulScan and videofluoroscopy (*r* = 0.686; *p* < 0.0001). Correlations between dynamic and functional imaging across runs also showed high reliability (mean correlation of first 3 latent variable timeseries was 0.49 (*p* < 0.001) within a run and 0.17 (*p* < 0.001) across runs), indicating that SimulScan with PLS can extract reliable maps of linked correlations between the brain and the oropharyngeal dynamics.

**Conclusion:**

The updated SimulScan with PLS analysis enables the study of central control of swallowing, providing simultaneous biomechanical visualization of the swallow along with brain functional signals.

## 1 Introduction

Swallowing is a complex function involving the precise coordination of oropharyngeal muscles, nerves, brainstem circuitry, and brain inputs [1]. Disruptions in these elements can lead to swallowing disorders (a.k.a. dysphagia), affecting about 9.44 million adults in the US annually, often due to neurologic conditions such as stroke and Parkinson's disease (PD) [2]. Neurogenic dysphagia is linked to poor outcomes such as reduced quality of life, malnutrition, and increased respiratory infections and mortality [3, 4, 5, 6, 7, 8]. A perplexing clinical challenge is that neurogenic dysphagia is highly heterogeneous even within a disease [9, 10], may result from involvement of many neural mechanisms [11, 12], and remains relatively poorly understood [9, 13]. This heterogeneity and limited understanding hinder the development of effective, physiology‐based swallowing treatments and inhibit patient outcomes.

To start addressing this gap and better understand the central control of human swallowing, studies have used a variety of neuroimaging methods, including task‐based functional MRI (fMRI). The vast majority of these studies have investigated healthy adults and have showcased an extensive bilateral network of cortical and subcortical areas, including the lateral primary somatosensory and motor cortex, the premotor area, the inferior frontal gyrus, as well as areas in the basal ganglia, the anterior cingulate, insula, cerebellum, and components of the brainstem (for a meta‐analysis, see [14]). Task‐based fMRI and resting state functional connectivity MRI (fc‐MRI) have also been increasingly used to examine the effects of neural disruptions, such as those caused by stroke and PD, on the larger sensorimotor network [15, 16, 17, 18], as well as to study swallowing neurophysiology in patient populations and brain plasticity related to treatments in patients with dysphagia [19, 20, 21, 22].

In studies using task‐based fMRI to examine swallowing control in stroke patients with dysphagia, results have been mixed, showing both hyperactivation and hypoactivation of key cortical areas compared to activity in healthy controls (e.g., [23, 24, 25, 26]). Pre‐post treatment/recovery studies have similarly provided preliminary data on changes in resting‐state or task‐based activity in specific brain regions that seem to be, at least partly, related to stroke size and severity [22, 27, 28, 29]. Although these studies have offered valuable initial information into central adaptations in neurogenic dysphagia, key challenges in applying this methodology have limited the number of participants and the overall number of studies in this field. These challenges primarily relate to the motion artifacts that are often introduced when patients swallow stimuli in the magnet, as well as the inability of patients with dysphagia to safely swallow under artificial conditions—for example, at exact times and with specific stimuli, as required for accurate fMRI imaging and analysis.

Furthermore, the task‐correlated motion and magnetic susceptibility changes during swallowing are a significant challenge for fMRI studies [30]. Swallowing involves motion of the oropharyngeal structures of interest, but can also be accompanied by unwanted bulk movement of the brain due to slight rotation of the head during a swallow. Due to spin history effects, this motion will show up as false fMRI activation that cannot be corrected through registration and motion correction approaches [31]. This can result in significant artifactual task correlations in the brain. The task‐correlated bulk motion artifacts usually occur around the periphery of brain structures where signal change will be highest from brain motion. Several strategies have been used to try to avoid this unwanted motion in swallowing studies. In [32], a behavioral interleaved gradient method [31] was used to place the swallowing motion in a quiet period (non‐imaging portion) of the functional MRI acquisition. This, however, makes the data acquisition less efficient with consistently longer TRs required to allow time for motion. Other approaches to minimize motion include adding extra head padding to minimize movement, monitoring motion during the scan to exclude data with excessive motion, and tracking precise timing of the swallow to attempt to separate motion and neural signals. In addition, there are navigator acquisitions that measure some physiological signal fluctuations with an additional acquisition to correct each functional image [33]. Despite these approaches, there is still a need for motion‐robust acquisitions that can capture swallowing motions and separate neural activations from motion‐induced signal fluctuations.

In order to understand the impact of central control on swallowing, there is a need to visualize the specific biomechanical deficits in swallowing function, as they provide critical information for diagnosis and treatment planning. In most prior fMRI studies, swallowing was monitored roughly through the use of monitoring devices such as surface electrodes or pneumographic belts in the neck area [32, 34, 35]. These methods can only indicate whether a swallow occurred without providing any diagnostic information on swallowing physiology or biomechanics. Further, they likely introduce sensory feedback, potentially impacting typical motor and sensory swallow function [36]. Current standard of practice for the diagnosis of dysphagia involves traditional imaging approaches like videofluoroscopy (real‐time x‐ray) and endoscopy, with both methods offering real‐time anatomical and physiological imaging of the swallow. Neither of these approaches enables the examination of the associated brain activity during the swallow to determine the impact of central control on a functional or dysfunctional swallow. Instead, to date, clinicians and researchers have been limited in acquiring brain activation information through MRI/fMRI or medical reports and swallowing imaging information from x‐ray/endoscopy separately and deriving relative correlations between the two (e.g., [11, 12, 21, 26, 37]). These mostly correlational attempts have left us with multiple unanswered questions about the specifics of the central swallowing control and have limited the development of personalized and effective treatments for neurogenic dysphagia [13, 38, 39].

The SimulScan (simultaneous acquisition of dynamic and functional MRI) sequence was introduced in order to address the need for fMRI of central control of swallows while simultaneously evaluating the biomechanics of those swallows and eliminating the need for a stimulus swallow in the supine position [36]. The sequence interleaved BOLD‐based fMRI with a dynamic imaging sequence to visualize motion associated with incidental swallows during a long, covert, fMRI study [36]. Although the sequence was able to achieve acquisition of fMRI and dynamic MRI, the dynamic data only had sufficient quality to visualize the timing onset of swallows for the covert swallowing task. The original implementation performed multishot dynamic imaging between each acquisition of a functional MRI slice, resulting in images that were highly distorted by magnetic susceptibility effects and had limited spatial coverage. Since publication of the SimulScan technique, new advances in low rank and Partial Separability (PS) models have enabled drastic increases in imaging speed, spatial resolution, and image quality [40]. These techniques have been used to create dynamic MR imaging studies that provide high‐quality, high temporal resolution images of speech and swallowing with image fidelity similar to structural scanning [41, 42, 43, 44, 45].

Given the potential for SimulScan to provide insights into neural control of swallowing changes with age and disease, an updated SimulScan imaging acquisition and reconstruction method are implemented that leverage recent advances in dynamic imaging, drastically improving the speed, image quality, and spatial coverage of the dynamic MRI portion of SimulScan. In addition, an analysis approach leveraging Partial Least Squares (PLS) is used to analyze the dynamic and functional neuroimaging data together in a data‐driven manner. We demonstrate that the new dynamic images from SimulScan can be reliably tracked through a Computational Analysis of Swallowing Mechanics (CASM) [46, 47] approach and are comparable to swallows acquired using the standard of practice tool, videofluoroscopy, in the same subjects. Finally, to demonstrate the reliability of the SimulScan method, we perform a test–retest experiment and analysis.

## 2 Methods

**FIGURE 1:**
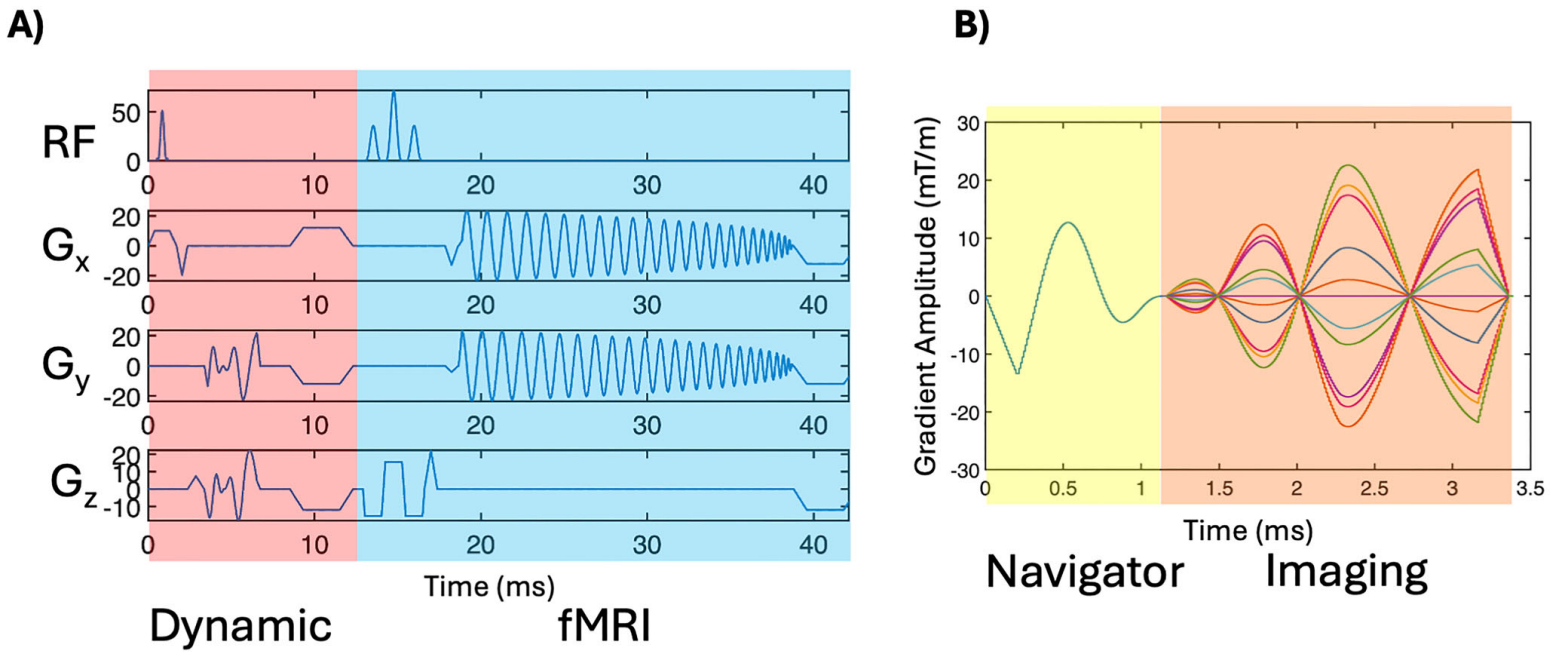
(A) Pulse sequence diagram for the SimulScan sequence. Red‐shaded portion is the sagittal dynamic imaging and blue shaded region is the 2D axial fMRI acquisition with TE of 25 ms. The fMRI acquisition uses water excitation from a binomial pulse and a spiral‐in acquisition. Gradients are in mT/m. (B) The dynamic imaging module (expanded to clearly display the navigator and imaging components) consists of a spiral‐in navigator that is repeated each time followed by a single shot of a multishot spiral‐out acquisition. Several shots are shown overlaid that would be used in different TR's.

*Updated Acquisition*: The SimulScan sequence (pulse sequence diagram in Figure 1A) interleaves a mid‐sagittal dynamic imaging sequence with a BOLD‐based fMRI acquisition collected with oblique axial slices in the brain. An overall TR (42.1 ms) includes a block for a sagittal dynamic acquisition (achieving 23.75 frames per second (fps)) and a block for a single slice of fMRI data (BOLD TR of 1.6 s for 38 slices). For the dynamic imaging portion of the sequence (Figure 1B), leveraging advances in PS‐model speech imaging [43, 44], we combine a spiral‐in temporal navigator that is repeated each dynamic block, followed by a single shot of an 80‐shot spiral‐out acquisition that rotates in each acquisition. This is immediately followed by a spiral‐in BOLD‐based fMRI acquisition with a TE of 25 ms acquiring 1 shot for a single 2D slice. We chose to combine the temporal navigator and imaging readout for the dynamic timeseries into the same excitation and readout to keep the frame rate as high as possible. This comes at the expense of a slightly increased echo time for the dynamic imaging data, which was seen to have minimal impact on visualizing the swallow. Compared to the original SimulScan sequence, the new 80‐shot acquisition for the dynamic imaging data has a readout duration of 2.2 ms (plus temporal navigator) compared to 19.8 ms that would be required per shot for the 6‐shot multi‐shot acquisition of the original SimulScan. This results in a much faster frame rate along with an order of magnitude reduction in magnetic susceptibility artifacts which are proportional to the readout duration [48]. See Video S1 for a comparison of the image distortions in the original SimulScan and the updated method.

In our imaging protocol, the SimulScan sequence acquired a dynamic image at 23.75 frames per second for a single mid‐sagittal 10‐mm thick slice. The spiral‐in temporal navigator was 1‐shot of a 40‐shot acquisition for a 30 cm FOV with a 3 mm spatial resolution, designed with a maximum gradient amplitude and slew of 23 mT/m and 120 T/m/s. The spiral‐out dynamic imaging data was a multishot spiral acquisition designed for a 30 cm FOV, 200 matrix size (but reconstructed at 225 for 1.3 mm in‐plane resolution), 80‐shot spiral with the same maximum gradient and slew rate, a 10° flip angle and both gradient and RF spoiling. Simultaneously, fMRI scans were taken for 38 slices, 3 mm thick, with in‐plane resolution 2.5 × 2.5 mm and matrix size of 96 × 96, acquired with a spiral‐in acquisition that was 1 shot of a 2‐shot acquisition, parallel imaging acceleration factor of 2, and a 90° flip angle binomial water excitation RF pulse, and a TR of 1.6 s for the fMRI scan.

From this acquisition, traditional 2D fMRI slices are reconstructed using a magnetic field inhomogeneity (field map measured in separate acquisition) corrected image reconstruction [49]. Dynamic data are reconstructed using the PS‐model reconstruction framework [43, 44]. Briefly, the spiral‐in navigator data are formed into a k‐space by time‐point matrix, and the top 40 right singular vectors are taken as the temporal basis. Then the spiral‐out dynamic imaging data are used to fit the spatial basis functions to the corresponding temporal basis through a least‐squares framework. The spatial basis functions are combined with the temporal basis functions to create a full timeseries of images.

### 2.1 Partial Least Squares

With the updated SimulScan sequence, we can achieve a rich set of dynamic imaging data and the associated brain activity underlying the swallows. To analyze central control of swallowing, we need to extract the brain activity related to the uncued swallows in the data. This requires a data driven approach to find dynamic relationships between the swallowing and fMRI data.

Partial Least Squares (PLS) has been used previously to analyze relationships between dynamic behavior and functional activations in fMRI data [50, 51]. In this method, a singular value decomposition (SVD) is used to extract latent variables of correlated components from the dynamic imaging and functional brain activation from a correlation matrix between the behavioral and fMRI data. Let the fMRI signals be stored in a matrix ***X***, which is a matrix that is *N*_*t*_ (number of total fMRI TR's in the SimulScan run) by *N*_*x*_ (the number of voxels in the 3D brain images). The brain extraction tool (bet) in FSL is used to provide a brain mask for ***X*** [52]. The dynamic imaging data will be stored in ***Y***, a matrix that is *N*_*t*_ by *N*_*y*_ (the number of voxels in the 2D mid‐sagittal dynamic imaging slice (or a region of interest in that slice)). We note that *N*_*t*_ must be the same between the dynamic imaging series and the fMRI data in our current implementation of PLS. To accomplish this, we let the dynamic information in ***Y*** be the temporal standard deviation in each voxel's magnitude across the fMRI TR, which is comprised of 38 dynamic images. This temporal standard deviation will show high signal in regions around moving edges in the dynamic images, providing a sensitive measure of motion. However, other measures of motion could also be used. Further, to account for the hemodynamic delay and response shape, we convolve all of the dynamic timeseries with a fixed hemodynamic response function (HRF), the canonical HRF from SPM (https://www.fil.ion.ucl.ac.uk/spm/software/spm12/) [53]. To summarize, the temporal standard deviation in the new functional TR windows was the dynamic signals comprising the matrix ***Y***.

We follow the formulation for the behavior approach for PLS [51], which is summarized here. For the brain imaging data, ***X***, each brain voxel's time course is zero‐meaned and normalized to unit standard deviation. However, in order to highlight motion events in the dynamic data, most of which are related to swallowing activity, we do not zero‐mean or normalize to unit variance the dynamic imaging data series, ***Y***. The correlation between the dynamic imaging data and the fMRI signals is formed by taking ***R*** = ***Y*** ^***T***^ ***X***. Taking the SVD of the correlation matrix, ***R***, results in a set of singular vectors, or saliences, ***U*** and ***V***. We choose a particular rank *L* to examine only the strongest correlated components. These singular vectors are the correlated spatial maps related to brain function (or functional activation maps for the correlated component) in the right singular vectors of ***R*** or the columns of ***V***, which is a *N*_*x*_ by *L* matrix. These can be thought of as the functional activation maps (brain function correlated components) for the dynamic motion represented in the same component of the spatial maps of the left singular vectors of ***R***, or the matrix ***U***, a *N*_*y*_ by *L* matrix. In order to see the time series of the activities of these components, we can extract temporal waveforms of correlated components by forming the latent variable timeseries as follows:

(1) $$L_{X}=\mathrm{XV},L_{Y}=\mathrm{YU}.$$

**FIGURE 2:**
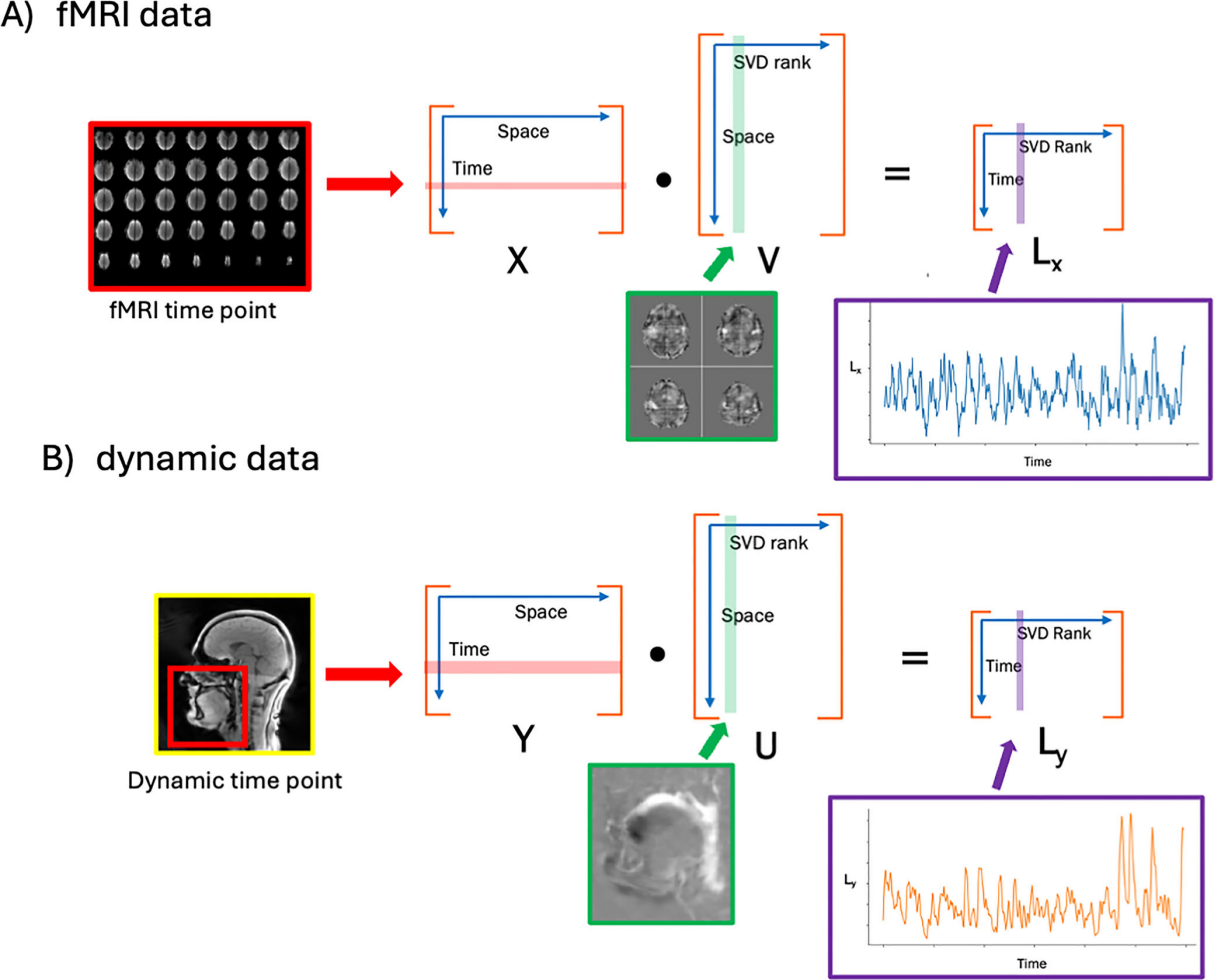
Depiction of the outputs of the behavioral partial least squares (PLS) approach, where the correlation, ***R***, is determined from the fMRI data, ***X***, and dynamic data, ***Y***, as ***R*** = ***Y*** ′ ***X***. (A) Layout of the fMRI data in matrix ***X***, and the matrix ***V*** extracted maps from the SVD of the correlation matrix, ***R***. One component of the spatial correlation maps in the columns of ***V*** is shown. This map is used to form the latent variable time series ***L***_***X***_. (B) Layout of the dynamic imaging data, ***Y***, showing the cropped region focusing on the oropharyngeal region in the red rectangle. The relationship is also shown between the dynamic data, ***Y***, and the spatial maps, ***U***, extracted from the SVD of the correlation matrix and its latent variable time series ***L***_***Y***_.

These latent variables, ***L***_***X***_ and ***L***_***Y***_, are *N*_*t*_ by *L*, giving the time series for each correlated component for the functional data and the dynamic data, respectively. Note that (Equation 1) represents applying each individual component's spatial map to the respective time series data to extract a weighted time series of that map's activity. See Figure 2 for an overview of the outputs of the PLS method.

Due to the large size of the data, custom Python libraries were written to provide the PLS analysis and other processing leveraging Dask (www.dask.org), a python library for parallel computing. This script is available on GitHub (https://github.com/mrfil/PLSCDemo.git). The MATLAB implementation of SVD of ***R*** requires loading the entire input matrix into memory and peak RAM usage during computation approached 10 times the size of the input, creating memory issues on a 256 GB RAM workstation. In contrast, the Dask implementation in python computes a randomly compressed rank‐*L* thin SVD, yielding only the user‐specified leading *L* singular values. In practice, the Dask implementation consumes approximately 20 times less RAM than the MATLAB implementation, enabling execution on a wide range of consumer hardware.

### 2.2 Evaluation of SimulScan

In order to evaluate the ability of the SimulScan sequence to capture dynamic motions associated with swallowing and to assess the reliability of fMRI components associated with swallowing, SimulScan data was acquired on 5 healthy young subjects (mean age = 25 ± 3.46 years; 3 female) in accordance with a protocol approved by the institutional review board at Purdue University (IRB# 2023‐714). All participants were healthy, in that they had no cognitive deficits based on a cognitive screening tool, Montreal Cognitive Assessment (MoCA) [54], and no swallowing concerns based on a swallowing screening tool, Eating Assessment Tool (EAT‐10) [55]. Participants were excluded if they had a history of head and neck cancer or neurological disease, or any condition which would contraindicate MRI. Data was acquired on a Siemens 3 T Prisma MRI scanner with a 64‐channel head coil at the Purdue Life Science MRI facility in West Lafayette, Indiana, using the SimulScan sequence along with T_1_ and T_2_ structural scans of the brain and oropharyngeal region.

Similar to our earlier work, a simple saliva swallowing paradigm was used while subjects watched a video of their choice. Four hundred and forty BOLD TR's were acquired per self‐paced swallowing SimulScan run, and two runs were acquired for each subject to allow test–retest examination of extracted correlated components. Each TR is 1.6 s so each run was 704 s or 11 min 44 s. For one subject, we acquired 600 BOLD TR's per run (Subject 1), but shortened to 440 for all other subjects. For each run, subjects were instructed to think of something tasty (to elicit saliva production) and swallow their saliva as frequently as comfortable throughout the acquisition, with no target number of swallows given, resulting in a variable number of swallows per run. Across all subjects, there was an average of 29 spontaneous swallows per scan. Subjects were padded into the head coil to minimize head motion and were instructed to keep their head still. Swallows were saliva swallows; no stimuli were given to the subject.

### 2.3 Dynamic Tracking Comparison Using CASM

Subjects who participated in the MRI experiment also performed swallows under videofluoroscopy (VFSS). VFSS was acquired using a videofluoroscopic C‐arm system (GE OEC 9800 Plus Digital Mobile 12 in.), which records images at 30 frames/s at the highest resolution (30 pulses/s). Subjects were placed in the supine position and performed 3 saliva swallows; however, these swallows were cued, which is necessary to reduce ionizing radiation exposure.

**FIGURE 3:**
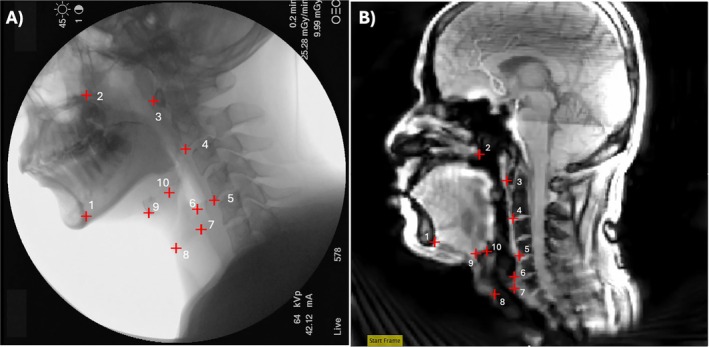
The landmarks used in Computational Analysis of Swallowing Mechanics (CASM). (A) The 10 anatomical landmarks tracked via CASM in VFSS. (B) The 10 anatomical landmarks tracked via CASM in dynamic MRI. Images have been rotated upright for the ease of the readers.

For the preliminary validation included herein, and given the large number of swallows completed during the SimulScan scans, we randomly selected and analyzed two swallows from the VFSS and two swallows from each SimulScan run for comparison. For both the dynamic MRI images and the VFSS swallows, CASM was performed using a semiautomated MATLAB tracking tool [56] to annotate 10 coordinates of anatomical landmarks mapping the functional anatomy of pharyngeal swallowing mechanics. Coordinates included five soft tissue landmarks (upper esophageal sphincter [UES], inferior edge of posterior vocal folds, inferior edge of anterior vocal folds, anterior inferior edge of hyoid bone [hyoid bone], and base of tongue/valleculae) and five hard tissue landmarks (inferior genial tubercle of mandible [mandible], intersection of posterior edge of hard palate and pterygoid plate of sphenoid bone [hard palate], anterior tubercle of first cervical vertebra [C1], anterior and inferior edge of the second cervical vertebra [C2], and anterior and inferior edge of fourth cervical vertebra [C4]) [46]. These landmarks are manually identified at swallow onset and then semi‐automatically tracked frame‐by‐frame to record coordinate data for multivariate morphometric analysis. Figure 3 displays the anatomical landmarks annotated on a frame of a VFSS and SimulScan, respectively.

All CASM raters met > 0.95 agreement with the method developer (author W.P.) on a training set prior to data analysis. Swallow videos were first trimmed to predefined start and end frames prior to CASM analysis [46, 57]. Because this study is the first application of CASM to saliva swallows, a new standardized trimming protocol was developed. Start and end frames for each image modality were determined differently to account for differences in temporal resolution and modality‐specific fidelity in visualizing anatomical structures. For VFSS, the first frame was defined as 3 frames before the velum starts elevating, and the end frame was defined as 10 frames after the hyoid bone starts moving posteriorly and inferiorly from its highest position. The start frame for SimulScan was defined as 7 frames before the first frame showing the onset of tongue base to posterior pharyngeal wall contact, and the end frame was defined as 3 frames after the last frame of tongue base to pharyngeal wall contact.

Matrix correlation analysis using Morpho J [58] was conducted to examine the correlations between the coordinates annotated in SimulScan and VFSS, as well as between the two SimulScan runs. After Procrustes superimposition of landmark coordinates in MorphoJ, covariance matrices describing landmark covariation were computed, and corresponding matrix elements were vectorized and compared using scatterplots to evaluate the similarity of covariance structure between datasets. Discriminant functional analysis (DFA) was used to further explore the landmarks that drive overall pharyngeal shape differences. DFA output was then imported into the MATLAB tracker tool and spatially adjusted using the C1, C2, and C4 vertebrae as anchor points to reorient the shape relative to fixed skeletal structures, enabling anatomically accurate visualization and interpretation [46].

### 2.4 Reproducibility Analysis

**FIGURE 4:**
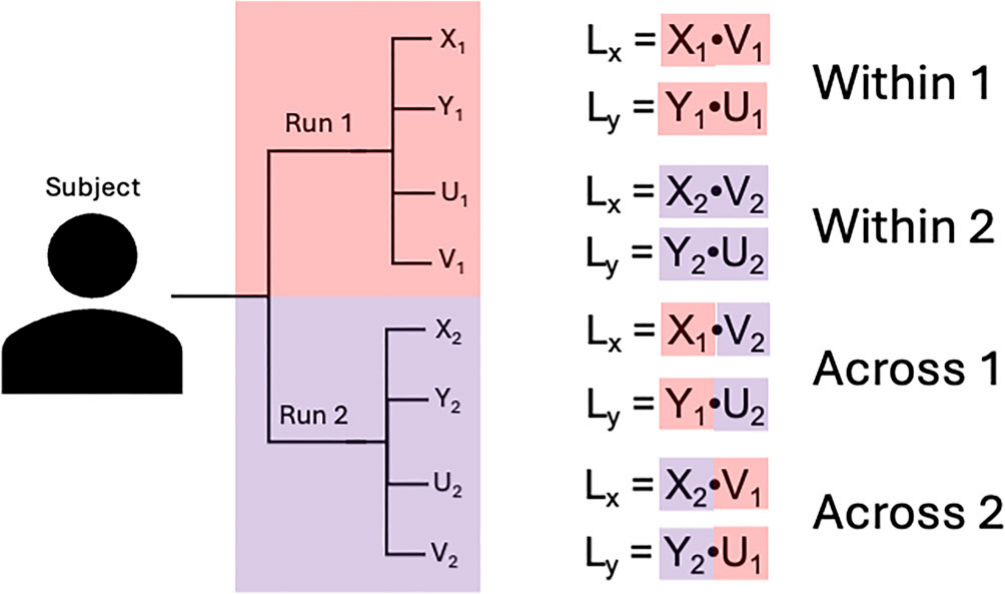
Schematic of test–retest comparison of Simulscan dynamic and functional imaging data. We form within run correlations, using maps and data from the same run, shown in the first 2 rows at the right. We also do cross‐run correlations using maps from 1 run and data from the other, shown in the bottom 2 rows.

To examine the reliability in extracting components of central control of swallowing, we leveraged the two runs of SimulScan in a test–retest analysis. In each run, the number and timing of swallows are different; however, if we are extracting functional regions involved in swallowing control, then we would expect that the same brain regions are correlated with the same regions of dynamic motion in the second scan compared to the first. For our test–retest analysis, we take the functional brain maps, ***V***, from run 1 and apply them to the functional brain imaging data, ***X***, in run 2. By doing this, we extract a cross‐run latent variable time series ***L***_***X***_ from run 2. We also take the dynamic heat map, ***U***, from run 1 and apply it to the dynamic imaging data, ***Y***, from run 2, forming a cross‐run latent variable time series ***L***_***Y***_ from run 2. If we have extracted correlated components, then the two latent variable time series we extracted with those masks should still be correlated. We examine this cross‐run latent variable correlation compared to within‐run correlation for all 4 pairings that are possible: two within run (data from run 1, maps from run 1; data from run 2, maps from run 2) and two across run (data from run 1, maps from run 2; data from run 2, maps from run 1). This is shown schematically in Figure 4. We use Pearson correlation between pairs of timeseries and a Fisher's *z*‐transform to average correlations.

## 3 Results

**FIGURE 5:**
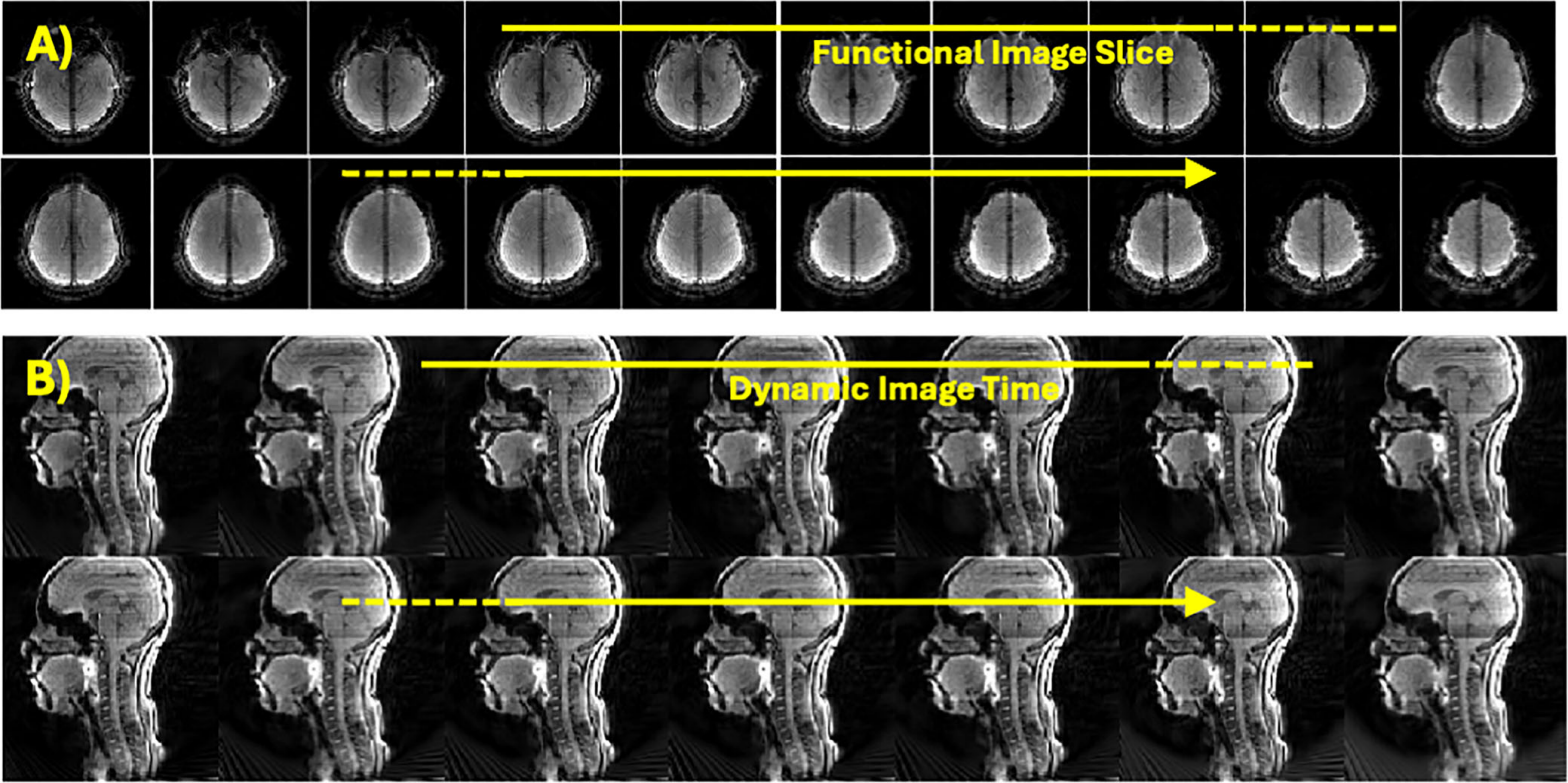
Example output from SimulScan. (A) Middle 20 slices shown from the BOLD fMRI scan showing the quality of the fMRI images. (B) 14 dynamic frames (589 ms) during a swallow in the same SimulScan run demonstrating the dynamic visualization of the swallow.

Figure 5 shows example images from the reconstruction of the SimulScan acquisition on a subject. In an 11 min 44 s scan, 440 functional volumes are acquired and 16,720 dynamic frames of the mid‐sagittal slice. Subjects were swallowing at their own pace and a time series of 14 images (589 ms) shows dynamic images of one of the swallows that occurred during the run (Figure 5B). The functional images show a dark line down the middle where the dynamic mid‐sagittal image is acquired. Likewise, in the brain region of the dynamic images, there are horizontal lines of the spin history effects of the acquisition of the axial slices for fMRI. These interactions are not in the functionally relevant portions of the images and will not impact the use of SimulScan for visualizing correlations between dynamic swallows and brain function.

**FIGURE 6:**
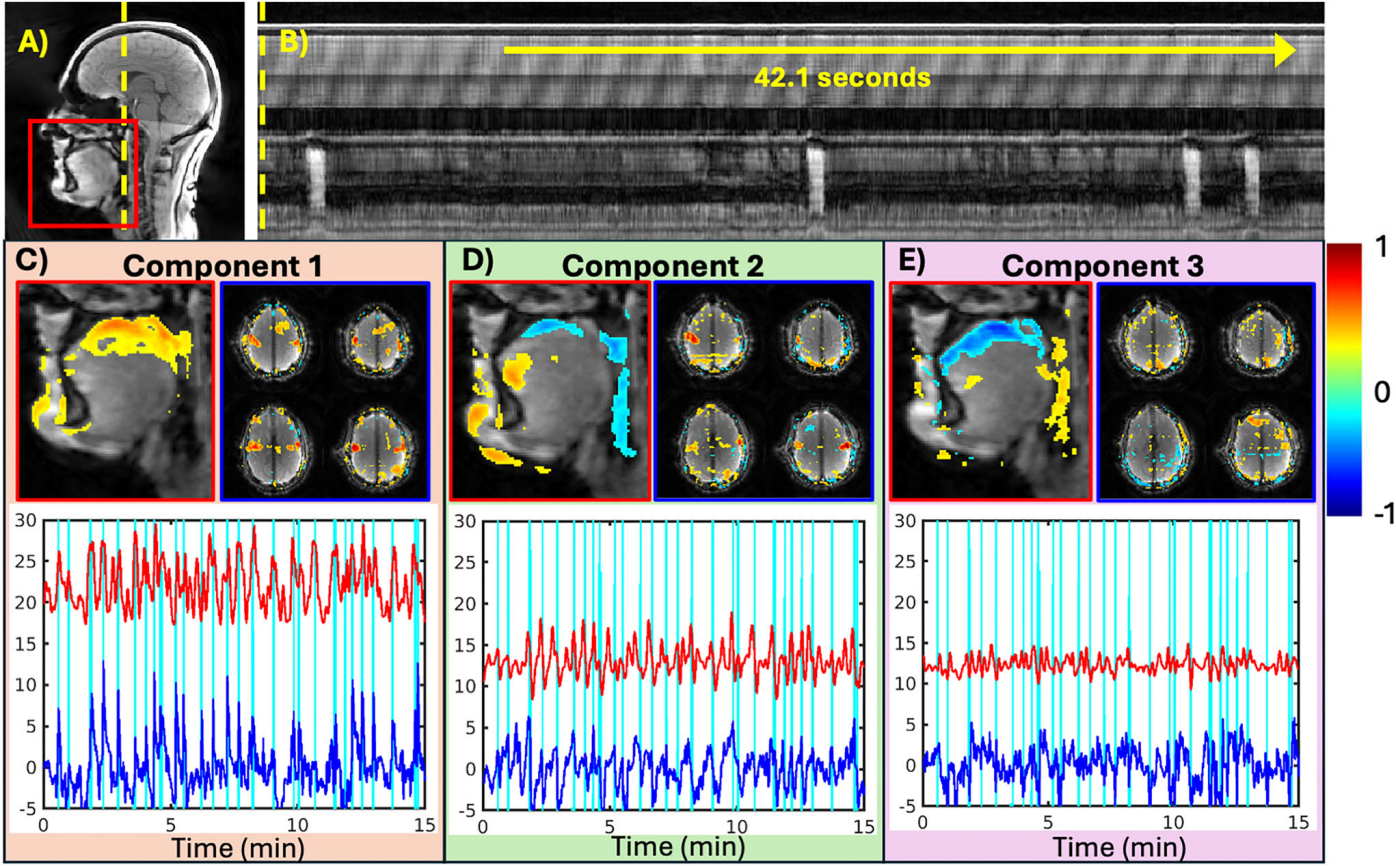
PLS components for one run in subject 1. (A) Mid‐sagittal dynamic image showing a red box to indicate the cropped image that went into PLS analysis. (B) A strip plot from the dotted line in panel (A) over 42 s of the acquisition showing 4 swallows that occurred during this time. (C) Output of PLS analysis for component 1. This shows the dynamic component overlaid on a reference dynamic image showing activity along the superior surface of the tongue. The functional MRI correlated maps are shown overlaid onto the fMRI images from SimulScan for 4 slices. This component shows lateral primary sensorimotor and anterior cingulate areas activated, as expected for the swallowing activity. The bottom timeseries plot shows the latent variable for the dynamic component (red) and the fMRI component (blue) along with the mean time series value (scaled) from a single pixel in the oropharyngeal port (cyan). The cyan line is to provide an indication where the signal intensity indicated a potential swallow. (D) Component 2 from the PLS analysis showing significant dynamic activity in the pharynx and also rotation of the jaw from bulk head motion. The fMRI activation map shows primary sensorimotor area activation along with some bulk motion artifacts. (E) Component 3 has reduced correlation between the dynamic component and the functional brain activations and shows functional signals around the perimeter of the head, demonstrating bulk motion artifacts from swallowing.

PLS analysis was performed on all runs from the study, extracting the top 3 correlated components. Figure 6 shows the components from Subject 1 that were extracted by PLS from within one run. In panels C–E, the activation or heat maps for the fMRI and dynamic data are shown with thresholding the largest magnitude (positive and negative) 10% of correlation intensities. Coefficient maps were each normalized by their maximum value before display. These masks are visualized as the colored overlays shown for both brain function and dynamic image slices, where 4 fMRI slices were extracted for ease of visualization. Note that components 1 and 2 (Figure 6C,D) show dynamic maps that represent the surface of the tongue and the pharynx motions during the swallow along with functional brain activation maps showing primary sensorimotor cortex and anterior cingulate activations associated with the oral/tongue and pharyngeal regions involved in swallowing [32]. There are some head motion artifacts in the functional image in component 2, seen as pixels around the edge of the head. The bottom part of panels C–E show the time series of the latent variable components for the dynamic (red) and the functional (blue) data from SimulScan. A cyan reference line is placed on the figure to indicate time points where a single pixel placed at the oropharyngeal port shows signal change, which may be indicative of a swallow occurring. Note that component 3 has less correlation between the dynamic and functional activations as seen in the latent variable timeseries, and shows mainly bulk head motion artifacts with activations around the edge of the brain. Similar maps for 3 other subjects (Subjects 2, 3, and 5) are shown in Figures S1–S3. Subject 4 is shown later in the fMRI analysis comparison.

**FIGURE 7:**
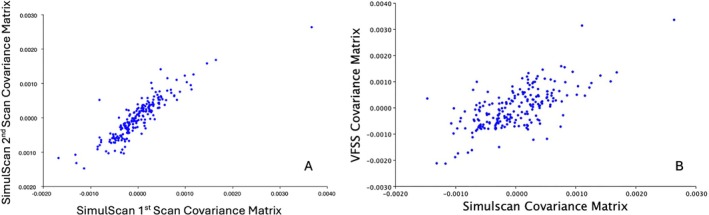
(A) Matrix correlation between first run of SimulScan and second run of SimulScan Procrustes covariance matrices. (B) Matrix correlation between SimulScan and VFSS Procrustes covariance matrices.

**FIGURE 8:**
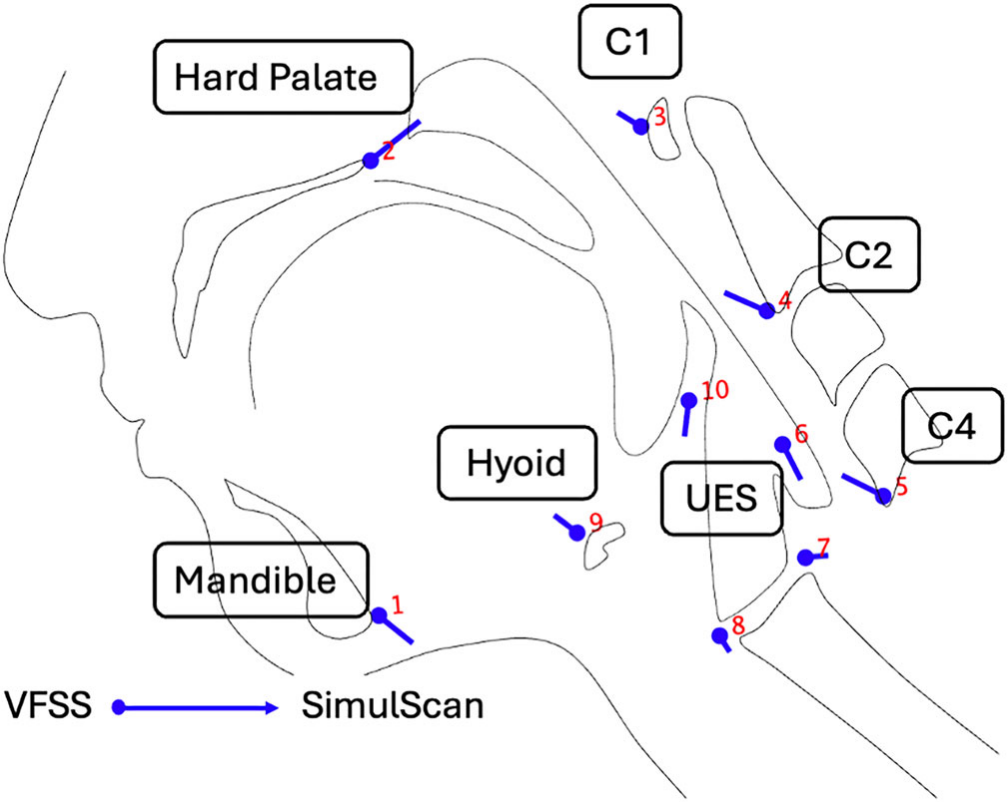
Eigenvectors from a discriminant function analysis illustrate greater differences in hard structures (mandible, hard palate, C1, C2, C4) and soft structures (hyoid bone, UES) when compared across imaging modalities.

In order to assess if the improved dynamic imaging from SimulScan is sufficient to track 10 key anatomical landmarks of the swallow biomechanics, CASM was applied to both the dynamic MRI and the VFSS data (Figure 7). A strong positive correlation was observed between the two SimulScan runs (*r* = 0.891; *p* < 0.0001; Figure 7A). Correlations between tracking measures for VFSS and dynamic MRI in this sample demonstrated a moderate positive correlation (*r* = 0.686, *p* < 0.0001; Figure 7B). We note that these analyses reflect different swallows as the VFSS and dynamic MRI are performed separately, but on the same day, with VFSS preceding the MRI scan for each subject. To further explore the landmarks' contributions to these results, discriminant functional analysis was used to compare differences in biomechanics reflected in these two modalities. In Figure 8, eigenvectors at each landmark reflect the direction and magnitude of mean variance of each coordinate. Larger magnitudes were observed with the five hard tissue landmarks, including mandible, hard palate, C1, C2, C4, and one soft tissue landmark, UES. The results indicate that these landmarks are driving the shape differences between the two modalities.

The component maps extract the correlated regions between brain function and dynamic motions during the swallow. To assess reliability of the correlations between the functional brain maps and the dynamic heat maps extracted by PLS, we examined correlations between the resulting latent variable timeseries for maps used both within and across runs. The correlations for all five subjects are given in Table 1 for the first 3 components, where most of the correlations are significant at *p* < 0.001 both in the within and across run conditions. Cross run correlations (taking the map from one run and applying it to the data of the other run) exceed 0.4 for two subjects. The mean correlation (using Fisher's *z*‐transform to average correlations) was 0.49 (*p* < 0.001) for the within run condition and 0.17 (*p* < 0.001) for the across run condition.

**TABLE 1:** Examination of reliability of dynamic and functional component maps from PLS analysis within and across runs.

		*X*_1_ *U*_1_–*Y*_1_ *V*_1_	*X*_2_ *U*_2_–*Y*_2_ *V*_2_	*X*_1_ *U*_2_–*Y*_1_ *V*_2_	*X*_2_ *U*_1_–*Y*_2_ *V*_1_
Subject (components 1–3)		_Within run 1_	_Within run 2_	_Cross run_	_Cross run_
Subject 1	C1	0.5234	0.4886	0.0670 x	0.1046 x
	C2	0.4671	0.5307	0.4472	0.3862
	C3	0.3609	0.4082	0.0723 x	0.1199*
Subject 2	C1	0.5789	0.6328	0.2780	0.3264
	C2	0.4315	0.4551	0.1950	0.3315
	C3	0.6995	0.4312	−0.1376*	−0.2436
Subject 3	C1	0.5212	0.5412	0.0638 x	0.3338
	C2	0.5819	0.4356	0.1743	−0.2198
	C3	0.5340	0.6008	0.1645	0.2854
Subject 4	C1	0.4941	0.4214	0.2277	0.2536
	C2	0.4215	0.3755	0.2117	0.1604
	C3	0.4301	0.3785	0.0350 x	−0.0052 x
Subject 5	C1	0.5234	0.4886	0.0670 x	0.1046 x
	C2	0.4671	0.5307	0.4472	0.3862
	C3	0.3609	0.4082	0.0723 x	0.1199*

*Note:* The table shows the correlation coefficient between the latent variable time course for the functional data, ***L***_***X***_, and the dynamic imaging data, ***L***_***Y***_, for cases in which the maps (***U***, ***V***) came from the same run or a different run as the data (***X***, ***Y***) of the SimulScan sequence. All correlations are significant at *p* < 0.001, except those marked. X indicates not‐significant, * indicates significant at *p* < 0.01 but not *p* < 0.001.

**FIGURE 9:**
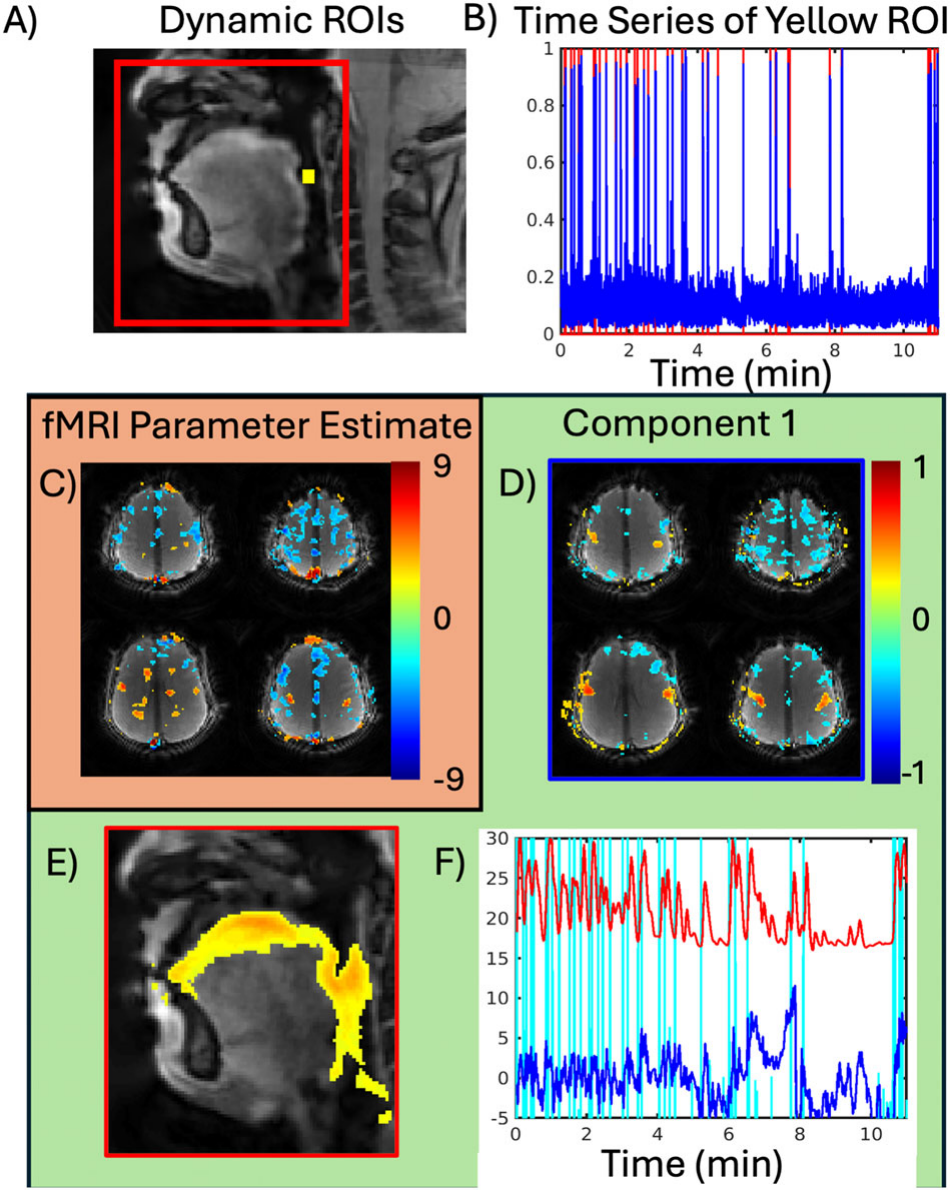
Comparison of standard fMRI and PLS results in self‐paced swallowing task in Subject 4. (A) Cropped dynamic image frame showing the yellow pixels as the region of interest for determining swallowing timing for standard fMRI analysis. The red box is the ROI used in PLS analysis. (B) Mean signal for the time series of the yellow pixels are shown in the blue trace and red lines are the detected swallows used in the timing of the task for the standard fMRI analysis. (C) *Z* statistic for the swallow task from standard fMRI analysis with GLM using swallow timings from B, only showing slices 8, 16, 22, and 26 for simplicity. (D–F) Component 1, from the PLS analysis of the (D) fMRI data (same slices as C) and (E) the dynamic component map. (F) The functional and dynamic component latent variable timeseries. Visualization of PLS component same as in Figure 6. Note that this component shows mostly functional activations with a little bulk motion artifact, especially at the top slice.

In order to further demonstrate the reliability and motion robustness of our analysis approach for the SimulScan data, we reanalyzed the SimulScan acquired data from one subject (Subject 4) with a traditional fMRI approach in FSL (6.0.5.1, [59]) including: motion correction using MCFLIRT [60]; spatial smoothing using a Gaussian kernel of FWHM 5 mm; and highpass temporal filtering (Gaussian‐weighted least‐squares straight line fitting, with sigma = 50.0 s). Automatic removal of motion artifacts using independent component analysis (ICA‐AROMA, [61]) was used to identify motion‐contaminated ICA components (49 out of 98 components flagged as motion in the data) which were then regressed from the data as confounds. ICA‐AROMA was run within the preprocessing pipeline of *fMRIPrep* 23.0.2 [62, 63], which is based on *Nipype* 1.8.6 [64]. We note that we could apply ICA‐AROMA to the PLS analysis also, but only applied it to the standard fMRI data to achieve similar ICA‐based motion removal to compare with the PLS analysis. Using the dynamic time series, we placed a control voxel in the posterior oropharyngeal region (see yellow pixels in Figure 9A) and extracted the time series. Since this region has tissue in it during a swallow, a high signal in this voxel's time series may indicate that a swallow is occurring. We used this timing as the task timing for a GLM analysis. With this analysis, we obtained the standard activation map shown in Figure 9C from the functional MRI images from our SimulScan acquisition, which has bulk motion corruption around the edge of the brain mixed with the activation signal despite ICA‐based fMRI motion correction. For comparison, we also include component 1 from the same SimulScan data demonstrating that SimulScan with PLS analysis can achieve robust brain activation maps despite task‐correlated motion, as seen in Figure 9D–F. Bulk motion components mostly separate out. Although there are some bulk motion related pixels around the brain in component 1, mostly at the top slice, the majority of bulk motion artifact shows up in later components.

## 4 Discussion

The updated SimulScan sequence achieves 23.75 fps dynamic imaging and a 1.6 s TR functional MRI to provide an unprecedented examination of the central control of swallowing. Importantly, the updated sequence integrates advances in fast dynamic imaging to produce clear images that provide significant information about swallow biomechanics. A strong positive correlation in tracking the 10 key anatomical landmarks over the swallow between two SimulScan runs shows that the dynamic MRI scan can reliably evaluate swallowing kinematics. Additionally, the correlation between VFSS and dynamic images from SimulScan demonstrated good agreement across modalities. Among the landmarks with greater magnitude difference, we believe that mandible, C1, and C4 likely reflect postural differences as the angle of the head laying down in the MRI magnet and during VFSS was slightly different. Although both were performed in the supine position, VFSS used a pillow that provided comfort to the subject, as seen in Figure 3. Differences related to hard palate, hyoid bone, and UES likely reflect differences in annotation strategies as each modality shows these structures differently [65]. However, in principle, these differences should not substantially affect within‐modality CASM analyses, which characterize the dynamic features of pharyngeal swallowing mechanics.

This analysis shows that the dynamic MRI scan can enable reliable evaluation of the functional anatomy underlying pharyngeal swallow mechanics. Although all subjects in the current study were healthy, this is an important early validation that provides confidence in the future use of this new sequence to evaluate disordered swallows in neurogenic dysphagia. With motion robustness and the use of saliva swallows, this technique has great potential for use with patient populations. SimulScan may enable the characterization of impacts of brain pathology on disruptions to swallowing function in stroke, Parkinson's Disease, and other clinical conditions, providing unparalleled insights into swallow deficits and plastic responses.

The PLS analysis for the SimulScan data was able to extract reliable correlated signals between the dynamic motions of the oropharyngeal region and brain function from the simultaneously acquired dynamic and functional imaging data. Subjects performed voluntary, uncued saliva swallows that vary throughout the scan. The PLS framework uses the dynamic information that is co‐acquired with the functional brain imaging data to find the correlated brain signals. In the current work, we characterized the dynamic motion through the temporal standard deviation in a pixel, highlighting large changes in signal that occur at the edges of the oropharyngeal structures during a swallow. Future work could incorporate other measures of motion for characterizing swallowing activity during the scan, focusing on biomechanical models of the swallow or kinematics. In addition, we note that subjects watched a video of their choosing during the acquisition. The functional activity from the movie or other activities would not be expected to correlate with the swallowing motion and would not be extracted as swallowing‐related brain activations.

For the PLS analysis in this work, we chose to zero‐mean and normalize the functional MRI data, but we kept the temporal standard deviation in the dynamic signal in its raw form. This formulation works as a feature extraction for the few swallows during the run with the dynamic signal maintaining a low value between swallows. In a dataset with significant head motion, this additional motion will result in non‐zero dynamic signals during rest. We expect that this motion will be extracted as separate correlated components with correlations appearing in the PLS component maps around the edge of structures, as was seen for the subject motion components in Figure 6 and Figures S1–S3. If several different types of motion occur, this could result in additional correlated components needed to represent this energy. In the current work, we used a rank of 3 in our PLS correlations, capturing over 92% of the energy of correlation in the first 3 ranks for each run of each subject. However, examination of the singular values of the correlation matrix can be used to determine if additional components are needed. Having insufficient rank could negatively impact the functional signal representation. Data with higher motion contamination will be examined in future work.

The PLS analysis routine on the SimulScan data also showed potential for robustness to bulk motion during the swallow, a challenge that plagues standard fMRI analysis approaches. Several PLS components showed the spatial pattern of correlated pixels around the complete edges of structures in both dynamic and fMRI images, clearly indicating that these are not neurological components. This approach at separating motion artifacts from functional activations is similar to other fMRI analysis methods which leverage the SVD or independent components to select components in fMRI data that are more related to neural activations instead of artifacts, such as in ICA‐AROMA [61]. Future work will examine how to automatically differentiate which components are significantly related to swallowing neural activity.

For the dynamic imaging with the PS model we need to acquire a temporal navigator for each dynamic imaging time point along with a single shot of imaging data (as shown in Figure 1). We have chosen to implement the temporal navigator and the imaging data in the readout with the same excitation pulse to achieve the fastest overall frame rate. This results in a slightly longer echo time for the dynamic imaging data, which could result in T2*‐induced signal loss in the image. The impact of this choice in the current swallowing study is minimal, but in a speech study with more air/tissue interfaces to disrupt the dynamic images, there will be a tradeoff between overall dynamic frame rate and image quality.

There are additional strategies that could be added to the analysis approach that may improve the results. First, no slice timing correction of the fMRI data in SimulScan was done in the current work. This fits with the strategy for the dynamic data in which we averaged the temporal standard deviation across the entire fMRI TR. Given the short fMRI TR of 1.6 s, slice timing is not expected to have a large impact on the functional maps correlated with the swallowing activity. However, this should be evaluated in future analyses, and we will explore leveraging the higher temporal resolution of the dynamic imaging data in the analysis. Second, BOLD‐based fMRI has a broad hemodynamic response function (HRF) that limits the ability to distinguish specific timing events in the swallow, which lasts less than 1 s. Therefore, the SimulScan with PLS approach presented here will not be able to separate components of the swallow dynamics and function based on timing. Instead, the PLS approach with the SVD operation creates spatially independent maps of swallowing that may separate components that vary significantly across different swallows during the timeseries. However, the main proposed use of the SimulScan method will be to compare differences in correlations between brain function and swallowing dynamics in healthy controls and neurogenic dysphagia patients. For these types of analyses, the main components of correlation between dynamic activity and brain function will be extracted across subjects in a group PLS analysis.

In the current work, we used a fixed HRF to model the coupling between dynamic motions and brain activation, leveraging the canonical HRF from SPM. In pathological conditions, this neurovascular coupling could be disrupted and may degrade estimations of the coupling between brain function and behavior [66].

In conclusion, an updated acquisition and analysis approach for SimulScan has significantly improved the quality and details of dynamic information available on the central control of swallowing. With this approach, we have achieved 23.75 fps dynamic imaging simultaneously with functional MRI of the brain at 1.6 s during self‐paced saliva swallowing tasks. Using PLS, correlated components can be extracted from the dynamics of the swallow and the brain activity during this task. This results in a motion‐robust analysis of swallowing related activity which has high test–retest reliability, as shown in a repeated scan framework. SimulScan has the potential to unlock studies of central control in swallowing dysfunction in neurogenic dysphagia and guide personalized treatment planning approaches in the future.

## Funding

This work was supported by the National Institutes of Health (R01AG078513, S10 OD012336).

## Conflicts of Interest

The authors declare that there are no commercial or financial relationships that could be construed as a potential conflicts of interest. The authors declare that this study received funding from the National Institute of Aging (Grant R01AG078513, M‐PIs: Sutton and Malandraki). Data acquisition was also supported in part by NIH grant S10 OD012336 (PI: Dydak).

## Supporting information


**Figure S1:** PLS results for Subject 2 with similar information as shown in Figure 6. (A) Midsagittal dynamic reference image indicating the region of interest for the dynamic component in the PLS analysis (red box). (B) Strip plot showing 2 swallows during 42 s of the acquisition. (C) Component 1 showing significant swallowing‐related activity in the dynamic image and correlated premotor and primary sensorimotor activations of the tongue/pharynx in the fMRI maps. There is some contamination with bulk motion artifacts around the edge of the brain. The latent variable timeseries follow the 10 swallowing events across the run. (D) Component 2 shows more varied activity in the dynamic map with voxels around the jaw and nose likely reflecting bulk motion from the swallow. The functional brain maps show similar motion‐related artifacts. (E) Likewise component 3 shows lower levels of correlation and components that have bulk motion related activity in both the dynamic and functional maps.
**Figure S2:** PLS results for Subject 3 with similar information as shown in Figure 6. (A) Midsagittal dynamic reference image indicating the region of interest for the dynamic component in the PLS analysis (red box). (B) Strip plot showing 3 swallows during 42 s of the acquisition. (C) Component 1 showing motion in the oropharyngeal area correlated with mostly bulk motion artifact around the brain in the functional image. This subject performed more swallows during the timeseries as seen in the latent variable timeseries plots. (D) Component 2 shows a mix between bulk motion artifacts as evidenced by activations around the head in the fMRI data but also shows some sensorimotor areas. (E) Component 3 shows oropharyngeal motion in the dynamic image and correlated premotor and lateral primary sensorimotor activations of the tongue/pharynx in the fMRI maps.
**Figure S3:** PLS results for Subject 5 with similar information as shown in Figure 6. (A) Midsagittal dynamic reference image indicating the region of interest for the dynamic component in the PLS analysis (red box). (B) Strip plot showing 3 swallows during 42 s of the acquisition. (C) Component 1 shows significant signs of bulk motion artifact with correlated pixels around the edges of structures in both the dynamic and fMRI data. (D) Component 2 shows dynamic activity in the pharynx with velar motion and primary motor cortex activity in the functional images, along with some bulk head motion artifact. (E) Component 3 shows a mix of bulk motion and pharyngeal activation in both the dynamic component and the fMRI map. Note that this subject shows significant numbers of swallows and a high degree of motion during the scan.

**Video S1:** A comparison of the image distortions in the original SimulScan and the updated method.

## Data Availability

The data that support the findings of this study are available on request from the corresponding author. The data are not publicly available due to privacy or ethical restrictions. Custom Python libraries were written to provide the PLS analysis and other processing leveraging Dask (www.dask.org), a python library for parallel computing. This script is available on GitHub (https://github.com/mrfil/PLSCDemo.git).
