# Supplementary material for: SimulScan and Partial Least Squares: Visualizing Swallowing Through Functional and Dynamic Imaging Correlations
Source: Magn Reson Med. 2026 Jun 21;96(4):1755–68. doi: 10.1002/mrm.70481 (PMC13419336; doi:10.1002/mrm.70481)
Supplement: Supplementary file 1 — Figure S1: PLS results for Subject 2 with similar information as shown in Figure 6. (A) Midsagittal dynamic reference image indicating the region of interest for the dynamic component in the PLS analysis (red box). (B) Strip plot showing 2 swallows during 42 s of the acquisition. (C) Component 1 showing significant swallowing‐related activity in the dynamic image and correlated premotor and primary sensorimotor activations of the tongue/pharynx in the fMRI maps. There is some contamination with bulk motion artifacts around the edge of the brain. The latent variable timeseries follow the 10 swallowing events across the run. (D) Component 2 shows more varied activity in the dynamic map with voxels around the jaw and nose likely reflecting bulk motion from the swallow. The functional brain maps show similar motion‐related artifacts. (E) Likewise component 3 shows lower levels of correlation and components that have bulk motion related activity in both the dynamic and functional maps. Figure S2: PLS results for Subject 3 with similar information as shown in Figure 6. (A) Midsagittal dynamic reference image indicating the region of interest for the dynamic component in the PLS analysis (red box). (B) Strip plot showing 3 swallows during 42 s of the acquisition. (C) Component 1 showing motion in the oropharyngeal area correlated with mostly bulk motion artifact around the brain in the functional image. This subject performed more swallows during the timeseries as seen in the latent variable timeseries plots. (D) Component 2 shows a mix between bulk motion artifacts as evidenced by activations around the head in the fMRI data but also shows some sensorimotor areas. (E) Component 3 shows oropharyngeal motion in the dynamic image and correlated premotor and lateral primary sensorimotor activations of the tongue/pharynx in the fMRI maps. Figure S3: PLS results for Subject 5 with similar information as shown in Figure 6. (A) Midsagittal dynamic reference image indicating the r [file MRM-96-1755-s002.pdf]

Supplementary figures for:

SimulScan and Partial Least Squares: Visualizing swallowing through functional and dynamic imaging correlations

Bradley P. Sutton[1,2,3], Anthony Bosshardt[1,3], Ching-Hsuan Peng[4], Ololade T. Adetula[2], Jiyoon Kim[1,2,5], Riwei Jin[1,5], Vaishnavi Krishna[4], William G. Pearson, Jr.[6], Zhongming Liu[7], Georgia A. Malandraki[4,8]

[1]Beckman Institute for Advanced Science and Technology, University of Illinois Urbana Champaign
[2]Grainger College of Engineering, Department of Bioengineering, University of Illinois Urbana Champaign
[3]Carle Illinois College of Medicine, University of Illinois Urbana Champaign
[4] Department of Speech, Language, & Hearing Sciences, College of Health and Human Sciences, Purdue University
[5]Coordinated Science Lab, University of Illinois Urbana Champaign
[6]Department of Biomedical Science, Via College of Osteopathic Medicine Auburn
[7]Department of Biomedical Engineering, Department of Electrical and Computer Engineering, University of Michigan Ann Arbor
[8]Department of Speech & Hearing Science, College of Applied Health Sciences, University of Illinois Urbana Champaign

Corresponding Author:
Brad Sutton, PhD.
Beckman Institute
405 N Mathews Ave
Urbana, IL 61801   bsutton@illinois.edu

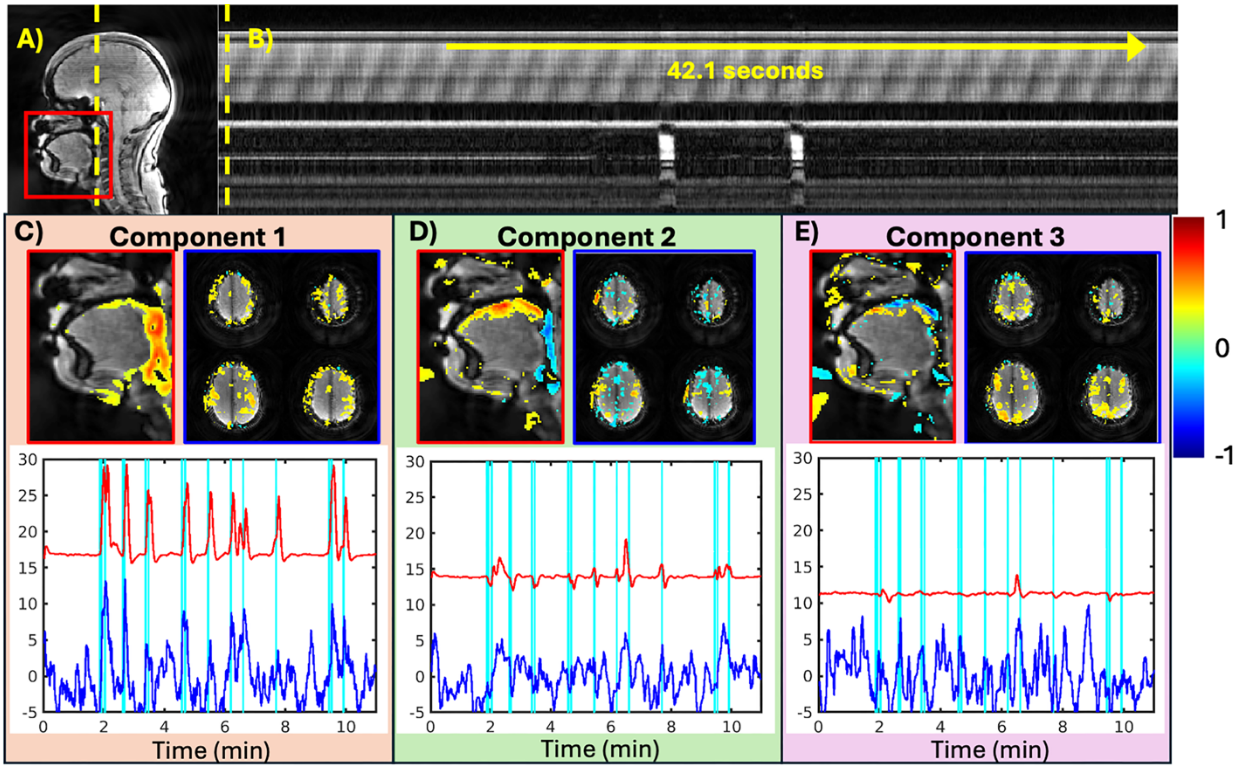

Supplementary Figure S1: PLS results for Subject 2 with similar information as shown in Figure 6. A) midsagittal dynamic reference image indicating the region of interest for the dynamic component in the PLS analysis (red box). B) Strip plot showing 2 swallows during 42 seconds of the acquisition. C) Component 1 showing significant swallowing-related activity in the dynamic image and correlated premotor and primary sensorimotor activations of the tongue/pharynx in the fMRI maps. There is some contamination with bulk motion artifacts around the edge of the brainThe latent variable timeseries follow the 10 swallowing events across the run. D) Component 2 shows more varied activity in the dynamic map with voxels around the jaw and nose likely reflecting bulk motion from the swallow. The functional brain maps show similar motion-related artifacts. E) Likewise component 3 shows lower levels of correlation and components that have bulk motion related activity in both the dynamic and functional maps.

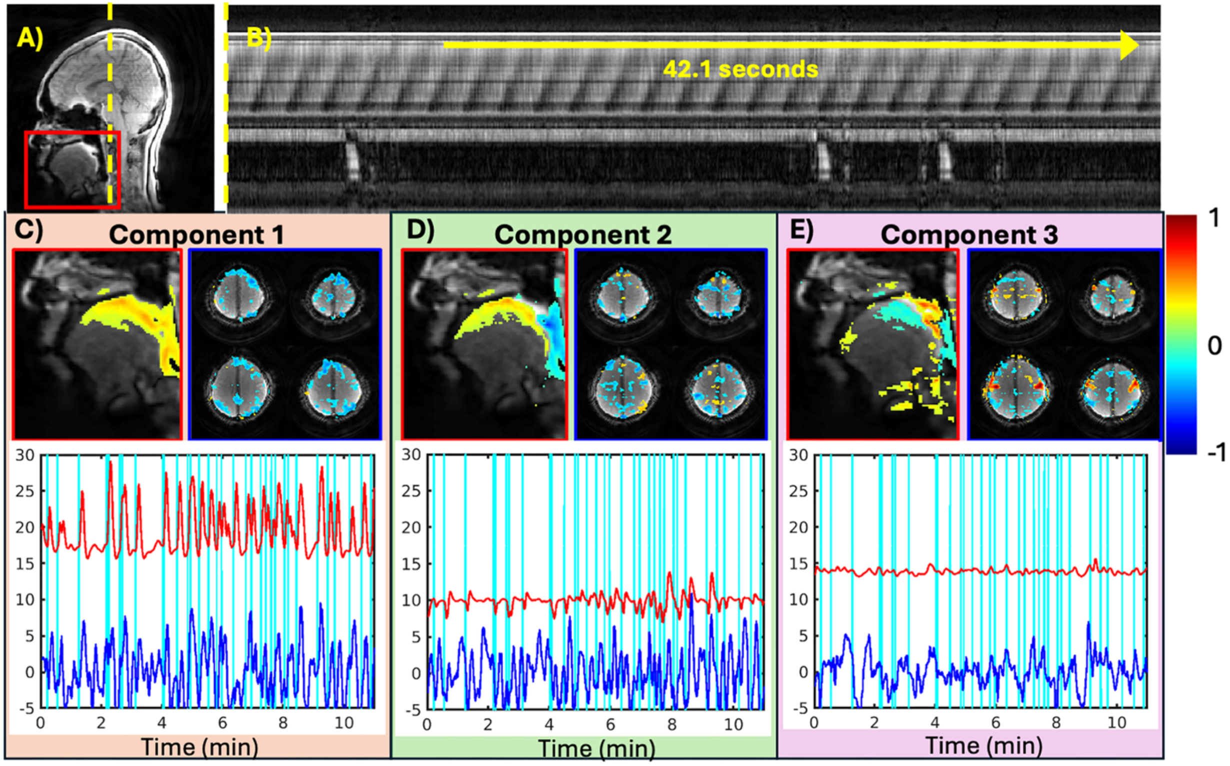

Supplementary Figure S2: PLS results for Subject 3 with similar information as shown in Figure 6. A) midsagittal dynamic reference image indicating the region of interest for the dynamic component in the PLS analysis (red box). B) Strip plot showing 3 swallows during 42 seconds of the acquisition. C) Component 1 showing motion in the oropharyngeal area correlated with mostly bulk motion artifact around the brain in the functional image. This subject performed more swallows during the timeseries as seen in the latent variable timeseries plots. D) Component 2 shows a mix between bulk motion artifacts as evidenced by activations around the head in the fMRI data but also shows some sensorimotor areas. E) Component 3 shows oropharyngeal motion in the dynamic image and correlated premotor and lateral primary sensorimotor activations of the tongue/pharynx in the fMRI maps.

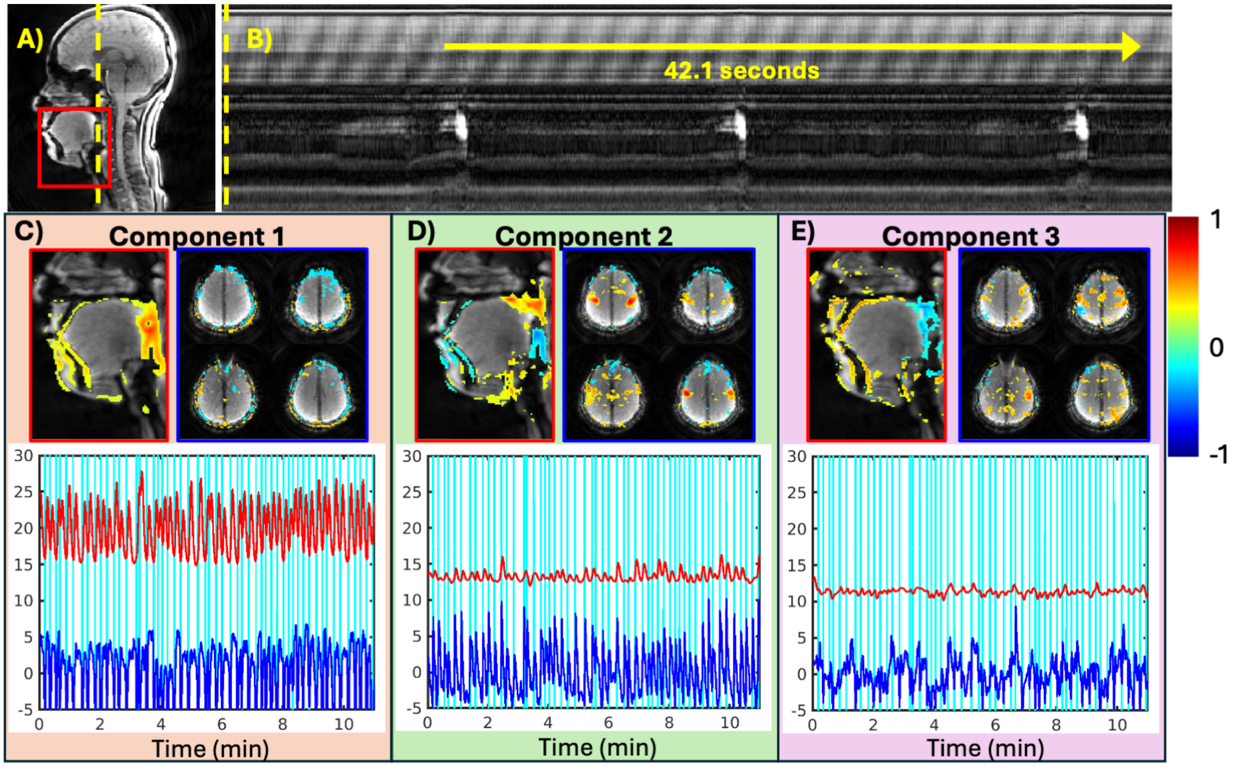

Supplementary Figure S3: PLS results for Subject 5 with similar information as shown in Figure 6. A) midsagittal dynamic reference image indicating the region of interest for the dynamic component in the PLS analysis (red box). B) Strip plot showing 3 swallows during 42 seconds of the acquisition. C) Component 1 shows significant signs of bulk motion artifact with correlated pixels around the edges of structures in both the dynamic and fMRI data. D) Component 2 shows dynamic activity in the pharynx with velar motion and primary motor cortex activity in the functional images, along with some bulk head motion artifact. E) Component 3 shows a mix of bulk motion and pharyngeal activation in both the dynamic component and the fMRI map. Note that this subject shows significant numbers of swallows and a high degree of motion during the scan.
